# Findings of preoperative patient risk factors to predict dislocation following total hip arthroplasty

**DOI:** 10.3389/fcell.2025.1601997

**Published:** 2025-05-15

**Authors:** Hao Li, Jinwei Xie, Xiaomin Lu, Shuai Li, Weiming Liao

**Affiliations:** ^1^ Department of Joint Surgery, The First Affiliated Hospital of Sun Yat-Sen University, Guangzhou, China; ^2^ Guangdong Provincial Key Laboratory of Orthopedics and Traumatology, The First Affiliated Hospital of Sun Yat-Sen University, Guangzhou, China; ^3^ Department of Orthopaedics, West China Hospital, Sichuan University, Chengdu, China

**Keywords:** total hip arthroplasty, postoperative dislocation, patient risk factors, risk factors for dislocation, preoperative planning

## Abstract

**Background:**

Previous studies have identified some risk factors of dislocation after total hip arthroplasty (THA). However, there are many vital preoperative patient risk factors remaining unknown. This study aimed to investigate comprehensively patient risk factors to reduce the dislocation rate after THA.

**Methods:**

We retrospectively reviewed patients who underwent primary THA in our department between January 2016 to December 2020. All readmissions related to postoperative dislocation were recorded, and each patient who dislocated was matched with three patients who did not dislocate according to date of operation, operative time and follow-up time. Patient risk factors were initially analyzed by univariate analyses, and independent risk factors for dislocation were identified by multivariate logistic regression.

**Results:**

A total of 5,133 patients were reviewed and 76 patients were readmitted with postoperative dislocations in follow-up time (1.5%). Age (odds ratio [OR], 1.027; 95% confidence interval [CI], 1.000–1.055; P = 0.049), rheumatoid arthritis (OR, 7.976; 95% CI, 1.419–44.827; P = 0.018), low serum calcium level (OR, 0.009; 95% CI, 0.000–0.211; P = 0.003) and poor education degree (OR, 0.847; 95% CI, 0.770–0.932; P = 0.001) were determined as independent predictors associated with dislocation after THA.

**Conclusion:**

Patients with older age, rheumatoid arthritis, low serum calcium level, and poor education degree require targeted optimization of preoperative planning and should be performed by appropriate surgical techniques and hip prostheses to prevent dislocation after THA and revision surgeries.

## Introduction

Total hip arthroplasty (THA) is a common surgical procedure used to treat end-stage joint diseases that may cause severe pain, deformities and bodily dysfunction (Mercurio et al., 2020; Saiz et al., 2019). Although the improvement of prosthesis design and surgical techniques has reduced the incidence of dislocation after THA to 0.05%–3.9%, the volume of THA is also on the rise and dislocation remains one of the most common reasons for failure and indication for early revision (Saiz et al., 2019; Dargel et al., 2014; Ding et al., 2020; Buckland et al., 2017; Malkani et al., 2010; Liu et al., 2019; Rowan et al., 2018). Most patients with a first dislocation have an increased factor for multiple occurrences, and subsequent treatment including reduction and revision THA, would bring higher charges to the patient and more complex surgery procedure to surgeons (Rowan et al., 2018; Kotwal et al., 2009; Abdel et al., 2015). Therefore, reducing the risk of dislocation after THA is essential to the orthopedists and the healthcare system (Saiz et al., 2019; Rowan et al., 2018).

In order to prevent of dislocation, risk factors for postoperative instability need to be identified (Rowan et al., 2018). Etiology of dislocation after primary THA is multifactorial, and risk factors are generally divided into four categories: patient risk factors, surgical techniques, implant design and management strategies (Saiz et al., 2019). The main risk factors of dislocation that have been identified are as follows: 1) patient risk factors: neuromuscular and cognitive disorders including cerebral palsy, Parkinson disease and dementia, cognitive dysfunction from aging, alcoholism and psychiatric diseases, prior hip fractures or surgical procedures, spinopelvic malalignment from spinal arthrodeses (fusions), degeneration or deformities, and decreased compliance (Dargel et al., 2014; Meek et al., 2006; von Knoch et al., 2002; Perfetti et al., 2017; Sing et al., 2016; DelSole et al., 2017; Kanawade et al., 2014; Zeng et al., 2023); 2) surgical techniques: less-experienced surgeons, posterior approach, unnormal femoral offset, component malposition, soft tissue imbalance, abnormal combined anteversion and prosthesis impingement (Charney et al., 2020; Forde et al., 2018; Lewinnek et al., 1978; Nakashima et al., 2014; Miki et al., 2013; Vaishya et al., 2015; Hedlundh et al., 1996; Zhang et al., 2015; Ji et al., 2012); 3) implant factors: without use of the elevated or constrained liner in patients considered to be at high risk for dislocation, smaller-diameter femoral head (Gill et al., 2016; Pace et al., 2015; Girard et al., 2013; Hailer et al., 2012; Howie et al., 2012; Tidermark et al., 2003; Munro et al., 2013); and 4) management strategies including lack of preoperative education to patients (Peter et al., 2011).

Although many risk factors of dislocation after THA are universally acknowledged, most previous studies were focused on the surgical techniques and implant factors such as component malposition and soft tissue imbalance, and it is obvious that there are some unknown factors involved (Liu et al., 2019; Dorr et al., 1983). Before surgeons perform the surgery procedures, detailing the patient risk factors of dislocation after primary THA could help them evaluate optimal surgical techniques and prosthesis selection to provide the best treatment for patients and decrease the likelihood of dislocations following primary THA (Saiz et al., 2019).

Therefore, based on the hypothesis that there are some unknown patient risk factors of dislocation after primary THA, we conducted a retrospective study aimed to determine undetected patient risk factors to reduce the rate of postoperative dislocation.

## Materials and methods

### Ethics statements

All procedures performed in studies involving human participants were in accordance with the ethical standards of the institutional and/or national research committee and with the 1964 Helsinki declaration and its later amendments or comparable ethical standards. The study was approved by the institutional review board. Informed consent and patients’ consent to participate were obtained from all participants.

### Cohort selection

All patients undergoing elective primary THA in our institution from January 2016 to December 2020 were reviewed retrospectively. Elective primary THA was defined by using International Classification of Disease-9th Edition (ICD-9) codes, and excluded patients with concomitant infection, hip fracture, tumor, or undergoing revision THA. A readmission that involved a hip dislocation or reduction of a hip dislocation was assessed as the primary outcome.

### Surgical technique

All primary THAs were performed via a posterolateral approach by five senior surgeons who performed more than 200 total hip arthroplasties per year. The patient was placed in the lateral decubitus position and the skin of the hip was incised through the fascia above the greater trochanter. The gluteus maximus was then divided and the external rotators were isolated. The specific procedures in the posterolateral approach were as described previously (Li et al., 2022). The acetabular implant was expected to be positioned in the “safe zone” of 40° ± 10° abduction and 15° ± 10° anteversion (Lewinnek et al., 1978). The hip capsule and external rotators were repaired. Senior surgeons explained the postoperative precautions to the patient and family in detail. After surgery, patients were encouraged to take limb exercises early, especially to strengthen hip abduction function exercises, and to perform partial weight-bearing walking for about 2 weeks, and gradually resume full weight-bearing walking 4–6 weeks after surgery. Each patient was followed up for at least 6 months after discharge.

### Statistical methods

All data management and statistical analyses were conducted using SPSS version 22.0 software (IBM Corp.). Continuous variables were calculated as means ± standard deviations, and they were analyzed using an independent Student’s t-test or a nonparametric test depending on normality of the data. Categorical variables were described as numbers and percentages. The Pearson chi-square test or Fisher exact test was used to analyze categorical variables according to whether the expected frequencies were greater than five. Univariate analyses were initially used to compare data between the dislocation group and the non-dislocation group. Then variables that had a p-value <0.1 in the univariate analyses were analyzed in a multivariate logistic regression analysis to determine independent patient risk factors for dislocation after THA. The significance level was set at p < 0.05.

## Results

In total, 5,360 primary THAs (5,174 patients) were performed in our department between January 2016 to December 2020. Among those patients, there were 19 patients were lost to follow-up and 22 mortalities during the follow-up period. Ultimately, a total of 5,133 patients were reviewed and 76 patients were readmitted with postoperative dislocations in follow-up time (1.5%). Twenty-six of those patients had more than 1 readmission for dislocation (0.5% of all patients undergoing elective THAs, 34.2% of patients who are readmitted for dislocation). Each patient who dislocated was matched with three patients who did not dislocate according to date of operation, operative time and follow-up time. Therefore, 76 patients who dislocated were included in this study, and 228 patients who did not dislocate were identified as controls.

Table 1 demonstrates the demographic characteristics of the patients. On average, patients in the dislocated group had an older age (60.2 ± 13.5) than those (53.2 ± 14.1) in the control group (P < 0.001). There was no significant difference in sex, height, weight, body mass index (BMI), body side and American Society of Anesthesiologists (ASA) status. The variables regarding indication, comorbidities and surgical history are summarized in Table 2. There was a significant difference (P < 0.001) between two groups in the indication for THA, revealing that the disease type is a dislocation risk factor. Moreover, the dislocated group had significantly higher proportion of patients with diabetes mellitus (14.5% vs. 7.0%, P = 0.048), neuromuscular disease (10.5% vs. 3.1%, P = 0.015) and previous hip surgery (17.1% vs. 7.5%, P = 0.015) than the control group. The preoperative laboratory values and social support of patients for risk evaluation are summarized in Table 3. Patients who dislocated had significantly lower levels of serum calcium (2.3 ± 0.1 vs. 2.4 ± 0.1, P < 0.001) and phosphorus (1.0 ± 0.2 vs. 1.1 ± 0.2, P = 0.006) than those who did not experience a dislocation. Additionally, patients in the dislocated group had lower education degree and poorer compliance with hip precautions during the postoperative period than patients in the control group (P < 0.001 and = 0.033, respectively).

**TABLE 1:** Demographic characteristics.

Indexes	Dislocated (n = 76)	Control (n = 228)	P Value
Age (years)^a^	60.2 ± 13.5	53.2 ± 14.1	<**0.001**
Female sex^b^	41 (53.9%)	119 (52.2%)	0.791
Height (cm)^a^	161.3 ± 8.6	160.9 ± 17.4	0.849
Weight (kg)^a^	62.1 ± 11.4	62.6 ± 12.7	0.772
BMI (kg/m^2^)^a^	23.9 ± 3.7	23.7 ± 4.1	0.779
Right side^b^	43 (56.6%)	113 (49.6%)	0.289
ASA status:1–2^b^	55 (72.4%)	154 (67.5%)	0.432

BMI, body mass index; ASA, american society of anesthesiologists.

^a^ Data are given as the mean ± standard deviation.

^b^ Data are given as the number (percentage) of patients.

The P value indicating a significant difference among groups is in bold.

**TABLE 2:** Indication, comorbidities and surgical history.

Indexes	Dislocated (n = 76)	Control (n = 228)	P Value
Indication for THA			<**0.001**
Osteonecrosis of femoral head^a^	35 (46.1%)	97 (42.5%)	
Primary or secondary osteoarthritis^a^	22 (28.9%)	104 (45.6%)	
Rheumatoid arthritis^a^	12 (15.8%)	5 (2.2%)	
Others^a^	7 (9.2%)	22 (9.6%)	
Hypertension^a^	19 (25.0%)	52 (22.8%)	0.696
Diabetes mellitus^a^	11 (14.5%)	16 (7.0%)	**0.048**
Respiratory disease^a^	6 (7.9%)	7 (3.1%)	0.097
Osteoporosis^a^	12 (15.8%)	19 (8.3%)	0.063
Neuromuscular disease^a^	8 (10.5%)	7 (3.1%)	**0.015**
Cognitive dysfunction^a^	6 (7.9%)	7 (3.1%)	0.097
Psychiatric disease^a^	4 (5.3%)	4 (1.8%)	0.111
Spinal fusion^a^	5 (6.6%)	5 (2.2%)	0.128
Previous hip surgery^a^	13 (17.1%)	17 (7.5%)	**0.015**
Opposite side arthrosis^a^	39 (51.3%)	125 (54.8%)	0.595
Opposite side THA^a^	22 (28.9%)	50 (21.9%)	0.213

THA, total hip arthroplasty.

^a^ Data are given as the number (percentage) of patients.

The P value indicating a significant difference among groups is in bold.

**TABLE 3:** Preoperative laboratory values and social support.

Indexes	Dislocated (n = 76)	Control (n = 228)	P Value
Preoperative anemia^a^	20 (26.3%)	44 (19.3%)	0.194
Preoperative hypoproteinemia^a^	8 (10.5%)	10 (4.4%)	0.087
Preoperative serum calcium (mmol/L)^b^	2.3 ± 0.1	2.4 ± 0.1	<**0.001**
Preoperative serum phosphorus (mmol/L)^b^	1.0 ± 0.2	1.1 ± 0.2	**0.006**
Discharge home^a^	43 (56.6%)	126 (55.3%)	0.842
Living alone^a^	54 (71.1%)	153 (67.1%)	0.523
Degree of education (years)^b^	6.8 ± 3.6	9.2 ± 4.0	<**0.001**
Non-compliance with hip precautions^a^	9 (11.8%)	11 (4.8%)	**0.033**

^a^ Data are given as the number (percentage) of patients.

^b^ Data are given as the mean ± standard deviation.

The P value indicating a significant difference among groups is in bold.

Then the multivariate logistic analysis was used to identify independent predictors of hip dislocation after THA (Table 4). Age was assessed and determined as an independent predictor associated with dislocation (odds ratio [OR], 1.027; 95% confidence interval [CI], 1.000–1.055; P = 0.049).The indication for the primary THA was a significant independent predictor of dislocation, as patients with rheumatoid arthritis had higher dislocation rates after adjusting for other variables (OR, 7.976; 95% CI, 1.419–44.827; P = 0.018). A lower serum calcium level was also related to increased risk of dislocation (OR, 0.009; 95% CI, 0.000–0.211; P = 0.003). Moreover, lower education degrees showed significantly higher risk of dislocation (OR, 0.847; 95% CI, 0.770–0.932; P = 0.001). Diabetes mellitus, respiratory disease, osteoporosis, neuromuscular disease, cognitive dysfunction, previous hip surgery, preoperative hypoproteinemia, serum phosphorus and non-compliance with hip precautions were not identified as independent risk factors of hip dislocation after THA in this multivariate logistic analysis.

**TABLE 4:** Multivariate analysis.

Indexes	OR estimate	Confidence interval	P Value
Age	1.027	1.000–1.055	**0.049**
Indication for THA			**0.019**
Osteonecrosis of femoral head	2.013	0.619–6.548	0.245
Primary or secondary osteoarthritis	0.966	0.288–3.238	0.955
Rheumatoid arthritis	7.976	1.419–44.827	**0.018**
Others	1	—	—
Diabetes mellitus	2.277	0.831–6.241	0.110
Respiratory disease	1.254	0.279–5.631	0.767
Osteoporosis	1.186	0.393–3.585	0.762
Neuromuscular disease	4.518	0.849–24.031	0.077
Cognitive dysfunction	1.477	0.239–9.139	0.675
Previous hip surgery	2.188	0.784–6.110	0.135
Preoperative hypoproteinemia	0.770	0.198–2.991	0.705
Preoperative serum calcium	0.009	0.000–0.211	**0.003**
Preoperative serum phosphorus	0.303	0.042–2.199	0.238
Degree of education	0.847	0.770–0.932	**0.001**
Non-compliance with hip precautions	0.641	0.174–2.361	0.504

OR, odds ratio; THA, Total hip arthroplasty. The P value indicating a significant independent predictor of dislocation is in bold.

## Discussion

Dislocation following primary THA is a well-known and potentially devastating complication (Wright-Chisem et al., 2022). Since the introduction of THA, orthopedists provided a number of strategies to prevent dislocation (Wera et al., 2012). While modern surgical techniques, hip prosthesis and postoperative care have attracted much attention, particular emphasis should be placed on preoperative planning in the prevention of dislocation. This study aimed to identify comprehensively patient risk factors to assist orthopedists in the preoperative planning to evaluate optimal surgical techniques and prosthesis to reduce the risk of postoperative dislocations. The most important finding of this study was that patients with older age, indication for rheumatoid arthritis, low serum calcium level and poor education degree had higher risk of dislocation following THA. These patients require targeted optimization of preoperative planning and should be performed by appropriate surgical techniques and hip prostheses to prevent dislocation after THA and revision surgeries.

The overall dislocation rate in this study was 1.5%, which is lower than the 2.84% reported by Goel et al., in 2015, which was limited in the Medicare population over the study period (1997–2011) (Goel et al., 2015). The lower dislocation rate in this study can be attributed to the improvement of surgical techniques and prosthesis, while the additional emphasis is still required to further eliminate the dislocation rate.

In the previous literature, older age was generally considered as an independent risk factor for hip dislocation (Ding et al., 2020; Malkani et al., 2010; Rowan et al., 2018; Jørgensen et al., 2014; Malkani et al., 2017). This result may be attributed to the weakness of the abductor muscles in the elderly (Ding et al., 2020; Falez et al., 2017). However, the cutoff age for postoperative THA dislocation is not consistent in the previous studies (Esposito et al., 2015). In a retrospective review of 22,097 THAs, the authors found that patients aged over 70 years had a significantly higher rate of dislocation than those aged 50–69 years (Esposito et al., 2015). Our study also found that patients who dislocated were older than those who did not dislocate.

Patients with rheumatoid arthritis have a greater demand for THA than those without rheumatoid arthritis because the disease is characterized by joint destruction and bone erosion (Zhou et al., 2022). Our study found that rheumatoid arthritis was an independent risk factor for dislocation after THA, which is consistent with the results of a previous study published in 2023 (Jiang et al., 2023). The study included patients undergoing THA for rheumatoid arthritis or osteoarthritis at their hospital between 2011 and 2021, and demonstrated that patients with rheumatoid arthritis showed significantly higher rates of wound aseptic complications, hip prosthesis dislocation, homologous transfusion, and albumin use. Patients with rheumatoid arthritis have a high prevalence of small femoral head, acetabular protrusion, suboptimal hip abductor strength and soft tissue laxity, which cannot provide adequate posterolateral support for the hip prosthesis (Ravi et al., 2014). Moreover, cortical thinning associated with long-term corticosteroid use and bone loss in patients with rheumatoid arthritis may be linked to increased incidence of hip prosthesis dislocation (Zhu et al., 2015).

The major finding of our study was the new identification of two risk factors for dislocation: low serum calcium level and poor education degree. Calcium is the core component of bone matrix mineralization, which is essential for bone formation and bone repair, and calcium deficiency may lead to osteoporosis and increase the risk of delayed fracture union, muscle spasms, periprosthetic osteolysis and aseptic loosening (Ciosek et al., 2021). However, maintenance of normal blood calcium levels is often neglected, even in patients diagnosed with osteoporosis. In a study including 505 patients with osteoporosis undergoing total joint arthroplasty, less than two-thirds of patients with osteoporosis or osteopenia received calcium or vitamin D treatments (Wang et al., 2022). In another study aimed at exploring the relationship between mortality and low bone mass density at the femoral neck and vertebra among patients self-discontinuing anti-osteoporosis medication, lower serum calcium levels were associated with higher mortality risk (Hsu et al., 2021). However, Okyaya et al. reported that serum calcium and ionized calcium levels were not associated with the development of osteoporosis (Okyay et al., 2013). Lower patient education degree is also an independent risk factor for dislocation after THA. Different from the lack of patient education and poor compliance with hip precautions during the postoperative period increasing the risk of dislocation (Dargel et al., 2014; Peter et al., 2011), low education levels make it difficult for patients to comprehensively understand the orthopedists’ instructions and inadvertently ignore the dislocation-prone movements. There is a significant relationship between knowledge level and education degree, but in a cross-sectional study involving 268 patients, the authors found that although adults with higher education levels had significantly more knowledge about healthy lifestyle habits than those with lower education levels, there was no significant relationship between education degree and osteoporosis lifestyle habits such as exercise, calcium and vitamin intake in patients’ daily lives (Etemadifar et al., 2013). In our study, after adjusting for other potential risk factors, osteoporosis was not an independent risk factor for dislocation after THA, so the reason why low blood calcium and poor education degree increase dislocation after THA may be unrelated to osteoporosis, and further research is needed to confirm our findings. For patients with low serum calcium level, orthopedists should pay attention to their bone density and guide them in calcium supplementation. For patients with low education levels, we need to explain to patients and their families in a more understandable way, such as drawing or animation, and actively communicate to prevent patients from doing actions that are prone to dislocation.

In order to reduce the dislocation rate after THA, orthopedists have made numerous attempts in surgical approach and prosthesis design. The posterolateral approach is a classic approach for THA, while great interest has been directed toward anterior approaches because of lower dislocation risk without increasing the risk of early revision (Sheth et al., 2015). Constrained liner is also an efficient way to reduce dislocation rate after THA. As displayed by Pace in a study, the impetus for using the constrained liner primarily was associated with significant decreases in the risk of dislocation following constrained THA (Pace et al., 2015). Therefore, for patients with the above patient risk factors of dislocation after primary THA, orthopedic surgeons can select appropriate approaches and prostheses to prevent dislocation.

The present study had several limitations. First, this is a retrospective study, which may lead to inevitable patient selection bias. In order to further thoroughly analyze the patient risk factors of dislocation after THA, more prospective studies with larger samples are needed in the future. Second, the indications for THA are not subdivided enough. For example, primary osteoarthritis and secondary osteoarthritis are classified into one category, and diseases other than osteoarthritis, femoral head necrosis and rheumatoid arthritis are collectively referred to as others. If the diseases are further subdivided, the interference of confounding factors may be further reduced, and more diseases may be identified as risk factors related to dislocation. Third, this study was a single-center study, which may reduce the generalizability of the research results. However, the generalizability will also be affected by different factors such as the center and individual. Therefore, the results in this study may be beneficial to extrapolate the results to a wider range of clinical practices involving different surgeons or settings. Finally, the muscles around the hip joint are easily affected by various factors such as diseases and age. However, the muscle strength was not compared due to the retrospective study design. In the future, more studies are needed to verify these theoretical hypotheses and further validate our results.

## Conclusion

Dislocation after THA remains a major challenge for orthopedists and is caused by multiple factors. Older age, rheumatoid arthritis, low serum calcium level, and poor education degree are independent risk factors for hip dislocation. Awareness of preoperative patient risk factors to predict dislocation can help orthopedists identify high-risk patients, make more accurate preoperative plans and select optimal surgical techniques and hip prostheses to prevent dislocation after THA and revision surgeries.

## Data Availability

The original contributions presented in the study are included in the article/supplementary material, further inquiries can be directed to the corresponding author.
